# Small-Gauge Pars Plana Vitrectomy for the Management of Symptomatic Posterior Vitreous Detachment after Phacoemulsification and Multifocal Intraocular Lens Implantation: A Pilot Study from the Pan-American Collaborative Retina Study Group

**DOI:** 10.1155/2015/156910

**Published:** 2015-10-04

**Authors:** Rodrigo M. Navarro, Leonardo M. Machado, Ossires Maia, Lihteh Wu, Michel E. Farah, Octaviano Magalhaes, J. Fernando Arevalo, Mauricio Maia

**Affiliations:** ^1^Brazilian Institute of Fight Against Blindness, 901 Otto Ribeiro, 19815-040 Assis, SP, Brazil; ^2^Department of Ophthalmology, Vitreoretinal Surgery Unit, Federal University of São Paulo, 822 Botucatu Street, 04023-062 São Paulo, SP, Brazil; ^3^Retina and Vitreous Service, Instituto de Cirugia Ocular, Costa Rica Diagonal a la Sala Garbo, Paseo Colón, P.O. BOX 3971-1000, San José, Costa Rica; ^4^Retina Division, Wilmer Eye Institute, Johns Hopkins University School of Medicine, 600 N Wolfe Street No. 327 Baltimore, MD 21287, USA

## Abstract

*Purpose.* To determine the efficacy of 23-gauge pars plana vitrectomy (PPV) for symptomatic posterior vitreous detachment (PVD) on visual acuity (VA) and quality after multifocal intraocular lenses (IOLs).* Methods*. In this prospective case series, patients who developed symptomatic PVD and were not satisfied with visual quality due to floaters and halos after multifocal IOL implantation underwent PPV. Examinations included LogMAR uncorrected visual acuity (UCVA), intraocular pressure, biomicroscopy, and indirect ophthalmoscopy at baseline and 1, 7, 30, and 180 days postoperatively. Ultrasonography and aberrometry were performed. The Visual Functioning Questionnaire 25 (VFQ-25) was administered preoperatively and at 30 days postoperatively. Both the postoperative UCVA and questionnaire results were compared to preoperative findings using the Wilcoxon test.* Results*. Sixteen eyes of 8 patients were included. VA significantly improved from 0.17 to 0.09 postoperatively (*P* = 0.017). All patients reported improvement of halos, glare, and floaters. VFQ-25 scores significantly improved in general vision (*P* = 0.023), near activities (*P* = 0.043), distance activities (*P* = 0.041), mental health (*P* = 0.011), role difficulties (*P* = 0.042), and driving (*P* = 0.016).* Conclusion*. PPV may increase UCVA and quality of vision in patients with bilateral multifocal IOLs and symptomatic PVD. Larger studies are advised.

## 1. Introduction

Posterior vitreous detachment (PVD) is defined as the separation of the posterior hyaloid from the internal limiting membrane [1]. It is age-related and becomes noticeable after the sixth decade of life (up to 63% prevalence) and is related to synchysis senilis [2]. Vitreous separation can cause visual symptoms such as photopsia from vitreoretinal traction and floaters resulting from the presence of condensed vitreous collagen [3]. About 30% of patients have floaters after development of PVD; however, most patients tolerate their symptoms [1, 2, 4]. A minority of patients report that these floaters are troublesome, in particular young myopic patients and those whose work requires detailed visual tasks. Pseudophakic patients frequently report floaters, which may be explained by the improved postoperative contrast sensitivity related to the intraocular monofocal lens, which increases the perception of floaters in the visual field [2, 4]. PVD incidence is also increased after cataract surgery by phacoemulsification with implantation of a posterior chamber intraocular lens (IOL) [4].

Multifocal IOLs were designed to reduce the need for spectacles by providing two or more points of focus. Adverse effects include reduced contrast sensitivity and the subjective experience of halos around lights [5].

The apodized diffractive multifocal AcrySof ReSTOR IOL (Alcon, Fort Worth, TX) was designed specifically to reduce glare and halos and provide increased dominant distance vision for patients with large pupils [5]. This lens has a central 3.6 mm apodized optic area with 12 concentric diffractive zones on the anterior surface for gradual reduction of the diffractive increments from the center to the periphery [6]. Rayner M-flex multifocal IOLs (Rayner, London, UK) are based on multizoned refractive aspheric optic technology, with either four or five annular zones (depending on the IOL base power [7, 8]).

The multifocal IOL design with concentric rings of optical zones creates positive dysphotopsias, also called photic phenomena. Visual phenomena interfering with vision strongly affect patient satisfaction [9]. Tolerance to visual phenomena caused by multifocal IOLs usually improves over time. Researchers believe the brain adjusts to the altered visual input over time through neural adaptation [9]. To experience the full visual benefits of multifocal IOLs, most patients require a neuroadaptation period of about 6 months [10, 11].

In the present study, the authors investigated the role of sutureless pars plana vitrectomy treatment in patients with symptomatic PVD in terms of visual acuity (VA) and visual satisfaction (measured through a standardized questionnaire) in patients who previously underwent bilateral implantation of the ReSTOR +3 (Alcon, Fort Worth, TX) or the M-Flex IOLs (Rayner, London, UK).

## 2. Methods

This prospective case series included patients who were not satisfied with their VA and complained of halos, glare, and floaters for at least 6 months after having undergone bilateral phacoemulsification using a 2.2 mm microincision technique and implantation of ReSTOR +3 (Alcon, Fort Worth, TX) or M-Flex IOLs (Rayner, London, UK).

Patients underwent a complete preoperative ophthalmologic examination including evaluation of refractive status, measurement of far uncorrected VA (UCVA) and best-corrected VA (BCVA), slit-lamp examination, Goldmann applanation tonometry, and indirect ophthalmoscopy. Ultrasonography, automated visual field measurement using the Humphrey 750i Visual Field Analyzer (Zeiss, Germany), and optical coherence tomography (OCT, Spectralis OCT, Heidelberg, Germany) were also performed, as well as a corneal aberrometry measurement (Galilei Dual Scheimpflug Analyzer, Ziemer Ophthalmology, Switzerland). The National Eye Institute Visual Function Questionnaire 25 (NEI VFQ-25) was administered before and 30 days after pars plana vitrectomy (PPV). All eyes were submitted to YAG-laser capsulotomy (Alcon, USA) at distinct periods after cataract surgery. Patients were informed about the possibility of being submitted to a sutureless PPV surgery to clear media opacities to obtain better focus of the multifocal IOL on the retina. All patients provided informed consent for sutureless 23-gauge PPV assisted by triamcinolone acetonide. The study protocol was approved by the Ethics Committees of the Federal University of São Paulo. The study was conducted according to the Declaration of Helsinki.

Patients were evaluated on postoperative days 1 and 7 and at 1, 3, and 6 months postoperatively. Ultrasonography was repeated at 7 days and 1 month after surgery. A new aberrometry was performed 6 months postoperatively.

Patients were included in the study if they were previously submitted to cataract surgery with multifocal IOL implantation and had been diagnosed with symptomatic PVD of at least 6 months of duration. Symptomatic PVD was defined as PVD detected by indirect ophthalmoscopy and fundus biomicroscopy revealing vitreous debris (which could include a Weiss Ring but that was not mandatory) in a patient with PVD-related visual symptoms (floaters and/or photopsias). Additionally, ultrasonography had to disclose PVD in the axial, temporal, nasal, inferior, and superior planes. The refractive error based on objective and subjective dynamic refraction could not exceed ±0.25 of spherical and no cylindric refractive errors. Patients also had to have aberrometry showing a root mean square (RMS) of less than 1.2 in both eyes. In addition, macular/retinal diseases had to be ruled out by spectral-domain OCT, fluorescein angiography, and automated visual fields (patients excluded if mean deviation values were not between +1.00 and −7.00 decibels).

Exclusion criteria were diabetes mellitus, age less than 45 years, a previous stroke or neurosurgical procedure, glaucoma or uveitis, previous ocular surgery other than cataract and multifocal IOL implantation, intraoperative complications during a previous phacoemulsification such as capsular tears, previous corneal diseases or scars, irregular corneal astigmatism, iris abnormalities, macular degeneration, neuroophthalmic disease, and postoperative complications such as macular edema and retinal detachment.

Vitreoretinal surgeries were performed by the same experienced surgeon (MM). Four-port PPV using three 23-gauge valved trocars (DORC, Amsterdam, Netherlands) was performed. A Tornambe (Synergetics, Missouri, USA) chandelier light pipe connected to a Photon II light source (Synergetics, MO, USA) was inserted into an additional 25-gauge sclerotomy. The surgical procedure was performed using the Stellaris PC vitrectomy system (Bausch & Lomb, USA) and a standard 23-gauge vitrectomy probe and the binocular indirect ophthalmomicroscope (BIOM) (Oculus, Germany) for visualization of the vitreous cavity. A core vitrectomy was performed using 5,000 cuts/minute and an aspiration rate of 200 mmHg for approximately 5 minutes followed by central posterior capsulectomy. In all cases, a flush of 0,3 mL of triamcinolone acetonide 4 mg/mL (Ophthalmos, Brazil) was used to confirm the posterior hyaloid removal; if still present, it was detached by direct aspiration with the vitrectomy probe with a vacuum rate set at 500 mmHg. Scleral indentation was performed and aspiration was reset at 200 mmHg for complete removal of the vitreous base and identification of possible retinal breaks. In bilateral cases, the same surgical technique was used 7 days later to treat the fellow eye.

The questionnaire of vision quality (NEI VFQ-25) was applied before and after PPV, in a 1-month interval. NEI VFQ-25 includes 25 questions that measure different components of visual function, with six additional optional items that enhance the reliability of the near and distance activity subscales. These were included in the Minimally Classic/Occult Trial of the Anti-VEGF Antibody Ranibizumab in the Treatment of Neovascular AMD (age-related macular degeneration) study and Anti-VEGF Antibody for the Treatment of Predominantly Classic Choroidal Neovascularization in AMD clinical trial [12]. The scores ranged from 0 (worst vision) to 100 (best, perfect vision-related function). There are 12 subscales: one general health subscale and 11 vision subscales, including general vision, difficulties related to near and distance vision activities, difficulties related to driving, vision-specific dependency, social functioning, role difficulties, limitations in peripheral and color vision, ocular pain, and mental health issues related to vision. The overall composite score was calculated using the mean of the subscales, excluding the general health subscale.

A Portuguese version of the questionnaire was applied and reliability was assessed using Cronbach's alpha coefficient, intraclass correlation coefficient, and interrater reliability coefficient. To estimate the power of the test, we considered simple random sampling and normal distribution for the VFQ-25 subscale scores. Given these assumptions, the estimated power of the test was between 50 and 90%, depending on the subscale [13].

The preoperative and postoperative NEI VFQ-25 scores and UCVA using logarithm of the minimum angle of resolution (LogMAR) charts were compared using the nonparametric Wilcoxon test. *P* < 0.05 was considered significant. The analysis was performed using SPSS software v. 21.0 (IBM, New York, USA).

## 3. Results

Sixteen eyes of eight patients were included in the analysis and all patients had improvement in UCVA after vitrectomy. All studied eyes were submitted to previous YAG-laser capsulotomy during the 6-month evaluation before the vitrectomy. Baseline data and UCVA values are shown in Table 1. All of the refraction values ranged from −0.25 to +0.25 diopters with no astigmatism preoperatively and did not change after PPV; hence, there were no differences between UCVA and BCVA values.  Table 2 shows the changes in the postoperative VFQ-25 scores on its subscales and the comparison between the preoperative and postoperative scores of the VFQ-25. The LogMAR UCVA levels are also presented (Tables 1 and 2).

**Figure 1:**
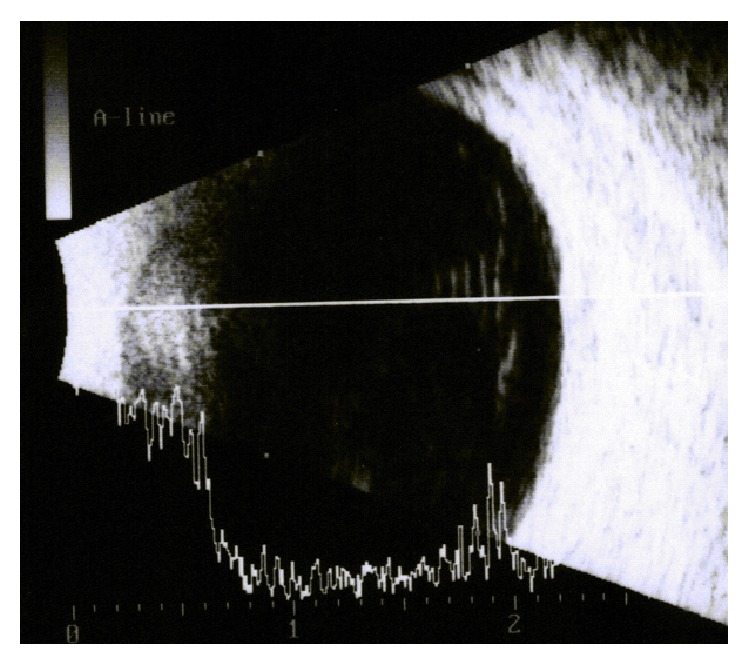
Example of ultrasonography image showing symptomatic PVD in one eye 7 months after multifocal IOL implantation in a patient complaining of floaters, halos, and poor quality of vision.

**Figure 2:**
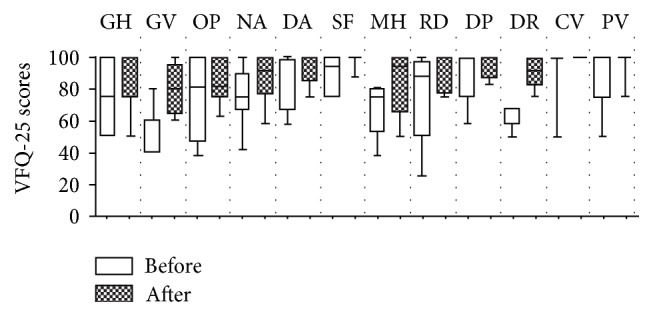
Boxplot of the preoperative and postoperative NEI VFQ-25 scores in the subscales of general health (GH), general vision (GV), ocular pain (OP), near activities (NA), distance activities (DA), social functioning (SF), mental health (MH), role difficulties (RD), dependency (DP), driving (DR), color vision (CV), and peripheral vision (PV).

VA significantly improved from a median value of 0.17 preoperatively to 0.09 postoperatively (*P* = 0.017) (Tables 1 and 2). All patients with symptomatic PVD confirmed by ultrasonography (Figure 1) who underwent PPV reported not only improvement of VA but also improvement regarding halos, glare, and floaters (each of these symptoms was reported preoperatively by all patients). The following postoperative NEI VFQ-25 subscales median scores significantly improved: general vision (from 60 preoperatively to 80 postoperatively, *P* = 0.023), near activities (from 75 to 92, *P* = 0.043), distance activities (from 67 to 100, *P* = 0.041), mental health (from 75 to 94, *P* = 0.011), role difficulties (from 88 to 100, *P* = 0.042), and driving (from 67 to 92, *P* = 0.016) (Table 2 and Figure 2).

At 6-month follow-up, there were no complications reported; specifically, no cases of rhegmatogenous retinal detachment and/or macular edema occurred in our series. Only 2 eyes underwent surgical induction of PVD. In the remaining 14 eyes, the posterior hyaloid was already detached at the time of surgery.

## 4. Discussion

**Figure 3:**
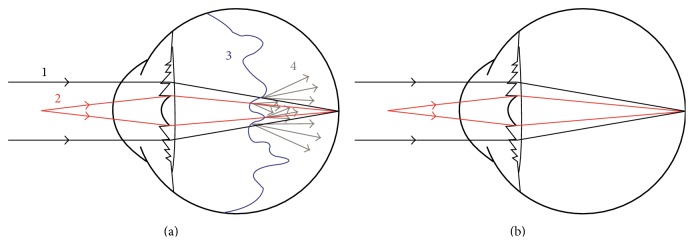
(a) Drawing showing an eye with a multifocal IOL and PVD. The light rays first pass through the cornea and then the IOL. The high density of the PVD causes dispersion of the light rays. (1) The light ray in black represents distance vision. (2) The light ray in red represents near vision. (3) The wavy blue line represents vitreous detachment. (4) The gray arrows show dispersion of the light rays when they pass through the dense vitreous causing halos, floaters, and blurred vision. (b) The drawing shows the light rays reaching the retina without interference from the vitreous detachment after PPV, indicating the potential for good near and distance vision without glasses. The red lines indicate the light rays for near vision.

Vitreous floaters are common symptoms that are classically treated by observation only [14]. Floaters may even be considered physiologic and age-related. However, they can be inconvenient and decrease the VA and visual quality of many patients [2]. These symptoms may be exacerbated by multifocal IOLs, since both PVD and these lenses are known to be responsible for increasing light scattering in ocular media [15, 16]. Diffractive multifocal IOLs generate two images simultaneously on the retina [17]. We hypothesize that symptomatic PVD associated light scattering [1, 18, 19] enhances this phenomenon by diffracting light rays in many directions thus worsening VA and quality of vision (Figure 3(a)). When there are no media opacities (such as after PPV), both near and far light rays can reach the retina at the same point of focus (Figure 3(b)).

PPV is a procedure that is usually reserved for complicated posterior segment disease. It has a well-known risk profile and, justifiably, there is reluctance to perform this surgery to treat floaters [2]. However, technological advances in both instrumentation and techniques have made PPV a much safer procedure [20, 21]. Small-gauge vitrectomy offers the advantages of minimal invasiveness, reduced postoperative inflammation and complications, and faster recovery [21].

The surgical management of floaters with PPV remains highly controversial among vitreoretinal surgeons. Plenty of evidence suggests that quality of life is improved in successfully operated patients [22–25]. The real issue remains, that is, the safety of this procedure [20, 26]. Induction of PVD during surgery is a major risk factor for the development of postoperative rhegmatogenous retinal detachment. In the current small series, we tried to include only eyes with complete PVD to reduce the risk of retinal detachment. However, even with preoperative ultrasonographic examination, 12.5% of eyes in our series still required intraoperative induction of PVD. No complications developed 6 months after vitrectomy. However, we should remain cautious given the small sample size and the relatively short follow-up. The authors think that the inclusion of only cases of bilateral disease was coincidental, or there is also the possibility that these patients have more intense symptoms related to PVD.

The results of the current study demonstrate the benefits of PPV in specific eyes with a multifocal IOL and symptomatic PVD. A statistically significant (*P* = 0.017) improvement in UCVA (Tables 1 and 2) was observed following PPV. The authors chose to report UCVA instead of the most common BCVA levels because usually patients with a multifocal IOL are expected to have a satisfactory good visual acuity without the need to use glasses or contact lenses; also, all patients presented no significant refractive error. All patients reported improvement regarding halos, glare, and floaters. To evaluate quality of vision, we compared the results of the VFQ-25 before and after vitrectomy in eyes that underwent previous bilateral multifocal IOL implantation (Table 2). The results showed significantly (*P* < 0.05 for all comparisons) improved postoperative VFQ-25 scores in all subscales tested (near and distance activities, mental health, role difficulty, and driving) (Table 2). A comparison between the VFQ-25 results in the pre- and postoperative periods at only 1-month interval after vitrectomy was performed in order to avoid the fact that binocular neurosensory adaptation could play a role in vision improvement; additionally at least 6 months was used as inclusion criteria since the initial symptoms, after the cataract surgery, until the indication of vitreoretinal surgery.

Some limitations of the current study must be addressed: first, the small number of patients enrolled, the short follow-up period, and absence of control subjects, since this was a pilot investigation; second, the lack of contrast sensitivity data, which was not included since the main objective was to observe the response of patients to surgery in terms of visual acuity and quality of life (as measured by the VFQ-25); third, the inclusion of posterior capsulotomy/capsulectomy in all eyes (capsulotomy at the preoperative period using the YAG-laser and capsulectomy during PPV). Previous capsulotomy was performed because capsular opacification may be related to the symptoms described [27]. Additionally, we hypothesized that residual capsular fragments or residual vitreous present at the posterior surface of the multifocal lens could also be related to these symptoms reported. Finally, the explanation of lack of wavefront analysis is based on the argument that we believe the clinical exam, the absence of astigmatism, and also the RMS value limit used (less than 1.2) are enough evidences to exclude that possible corneal problems, could be the cause of decrease in quality of vision.

Despite these, our preliminary results are exciting. To the best of our knowledge, this is the first study to address the importance of the posterior vitreous surgery in VA and quality of vision in eyes implanted with bilateral multifocal IOLs. On the basis of these preliminary data, it is reasonable to conclude that the status of the vitreous is important in patients who may undergo multifocal IOL implantation. Preoperative evaluation of candidates for these intraocular lenses implantations should include an assessment of the vitreous. Patients with symptomatic PVD who are not satisfied with their VA and quality of vision may benefit from small-gauge PPV in specific situations reported. However, additional clinical trials with large series of patients performed by different surgeons are necessary to confirm these preliminary observations.

## Figures and Tables

**Table 1:** Baseline patients' characteristics and uncorrected visual acuity testing in both eyes prior to and after 23-gauge sutureless pars plana vitrectomy.

Patient	Gender	Age (years)	Time interval between symptoms onset and PPV (months)	Eye	Preoperative UCVA (log⁡MAR)	Postoperative UCVA (log⁡MAR)	Preoperative corneal aberrometry (RMS)	Postoperative corneal aberrometry (RMS)	Preoperative OCT central foveal thickness (*μ*m)	Preoperative automated visual field MD (dB)
1	F	67	12	OD	0.17	0.17	1.1	1.1	226	−1.68
				OS	0.17	0.17	1.0	1.0	246	−1.54
2	M	58	12	OD	0.30	0.09	1.1	1.1	250	−1.0
				OS	0.30	0.09	1.1	1.0	227	−0.5
3	F	54	6	OD	0.09	0.00	0.8	0.7	260	−0.2
				OS	0.09	0.00	0.8	0.8	264	−0.4
4	M	44	8	OD	0.09	0.00	0.4	0.4	246	−1.0
				OS	0.09	0.00	0.6	0.5	249	−0.5
5	F	50	7	OD	0.30	0.17	0.4	0.3	232	+0.5
				OS	0.30	0.17	0.5	0.4	230	0.0
6	M	62	10	OD	0.09	0.09	1.0	1.0	228	−2.92
				OS	0.09	0.09	1.1	1.1	244	−2.32
7	M	64	12	OD	0.30	0.17	0.6	0.4	262	−0.54
				OS	0.30	0.17	0.4	0.4	248	−0.64
8	M	62	11	OD	0.17	0.09	1.0	0.9	196	−6.73
				OS	0.30	0.17	0.9	0.9	198	−5.72

RMS: root mean square.

MD: mean deviation.

**Table 2:** Results of the VFQ-25 subscales and uncorrected visual acuity (UCVA) used to compare the preoperative period to the postoperative one.

Parameter	Preoperative Median (range)	Postoperative Median (range)	*P* ^1^	*n* ^2^
General health	75 (50–100)	75 (50–100)	*0.157*	2 (25%)
General vision	60 (40–80)	80 (60–100)	*0.023* ^*∗*^	6 (75%)
Ocular pain	81 (38–100)	81 (63–100)	*0.180*	2 (25%)
Near activities	75 (42–100)	92 (58–100)	*0.043* ^*∗*^	5 (63%)
Distance activities	67 (58–100)	100 (75–100)	*0.041* ^*∗*^	5 (63%)
Social functioning	94 (75–100)	100 (88–100)	*0.083*	3 (38%)
Mental health	75 (38–81)	94 (50–100)	*0.011* ^*∗*^	8 (100%)
Role difficulties	88 (25–100)	100 (75–100)	*0.042* ^*∗*^	5 (63%)
Dependency	100 (58–100)	100 (83–100)	*0.102*	3 (38%)
Driving	67 (50–67)	92 (75–100)	*0.016* ^*∗*^	7 (100%)
Color vision	100 (50–100)	100 (100–100)	*0.317*	1 (13%)
Peripheral vision	75 (50–100)	100 (75–100)	*0.059*	4 (50%)

UCVA (log⁡MAR)	0.17 (0.09–0.30)	0.09 (0.00–0.17)	*0.017*	

^1^Wilcoxon test.

^2^Number of patients that got better scores after surgery.

UCVA: uncorrected visual acuity.

^*∗*^ *P* < 0.05.

## Summary Statement

Pars plana vitrectomy improves visual acuity and quality of vision in patients implanted with a multifocal intraocular lens who have clinically relevant posterior vitreous detachment and are not satisfied with their vision.

## References

[1] Foos R. Y. (1972). Posterior vitreous detachment. *Transactions—American Academy of Ophthalmology and Otolaryngology*.

[2] Foos R. J., Wheeler N. C. (1982). Vitreoretinal juncture. Synchysis senilis and posterior vitreous detachment. *Ophthalmology*.

[3] Coffee R. E., Westfall A. C., Davis G. H., Mieler W. F., Holz E. R. (2007). Symptomatic posterior vitreous detachment and the incidence of delayed retinal breaks: case series and meta-analysis. *American Journal of Ophthalmology*.

[4] Mirshahi A., Hoehn F., Lorenz K., Hattenbach L.-O. (2009). Incidence of posterior vitreous detachment after cataract surgery. *Journal of Cataract and Refractive Surgery*.

[5] Vega F., Alba-Bueno F., Millán M. S. (2011). Energy distribution between distance and near images in apodized diffractive multifocal intraocular lenses. *Investigative Ophthalmology & Visual Science*.

[6] Santhiago M. R., Netto M. V., Barreto J., Gomes B. A. F., Schaefer A., Kara-Junior N. (2010). Wavefront analysis and modulation transfer function of three multifocal intraocular lenses. *Indian Journal of Ophthalmology*.

[7] Prieto J. C., Bautista M. J. (2010). Visual outcomes after implantation of a refractive multifocal intraocular lens with a +3.00 D addition. *Journal of Cataract and Refractive Surgery*.

[8] Chang J. S. M., Ng J. C. M., Lau S. Y. F. (2012). Visual outcomes and patient satisfaction after presbyopic lens exchange with a diffractive multifocal intraocular lens. *Journal of Refractive Surgery*.

[9] Sood P., Woodward M. A. (2011). Patient acceptability of the tecnis multifocal intraocular lens. *Clinical Ophthalmology*.

[10] Bautista C. P., González D. C., Gómez A. C., Bescos J. A. C. (2009). Evolution of visual performance in 250 eyes implanted with Tecnis ZM900 multifocal IOL. *European Journal of Ophthalmology*.

[11] Yoshino M., Inoue M., Kitamura N., Bissen-Miyajima H. (2010). Diffractive multifocal intraocular lens interferes with intraoperative view. *Clinical Ophthalmology*.

[12] Suñer I. J., Kokame G. T., Yu E., Ward J., Dolan C., Bressler N. M. (2009). Responsiveness of NEI VFQ-25 to changes in visual acuity in neovascular AMD: validation studies from two phase 3 clinical trials. *Investigative Ophthalmology & Visual Science*.

[13] Simão L. M., Lana-Peixoto M. A., Araújo C. R., Moreira M. A., Teixeira A. L. (2008). The Brazilian version of the 25-Item National Eye Institute Visual Function Questionnaire: translation, reliability and validity. *Arquivos Brasileiros de Oftalmologia*.

[14] Martínez-Sanz F., Velarde J. I., Casuso P., Dez-Cotero J. N. F. (2009). Surgical solution to vitreous floaters visual problem. *Archivos de la Sociedad Española de Oftalmología*.

[15] Pinero D. P., Ortiz D., Alio J. L. (2010). Ocular scattering. *Optometry & Vision Science*.

[16] Langeslag M. J., van der Mooren M., Beiko G. H., Piers P. A. (2014). Impact of intraocular lens material and design on light scatter: in vitro study. *Journal of Cataract & Refractive Surgery*.

[17] Ji J., Huang X., Fan X., Luo M. (2013). Visual performance of acrysof ReSTOR compared with a monofocal intraocular lens following implantation in cataract surgery. *Experimental and Therapeutic Medicine*.

[18] Kanski J. J. (1975). Complications of acute posterior vitreous detachment. *The American Journal of Ophthalmology*.

[19] Castilla-Marti M., van den Berg T. J. T. P., de Smet M. D. (2015). Effect of vitreous opacities on straylight measurements. *Retina*.

[20] Wilkinson C. P. (2011). Safety of vitrectomy for floaters—how safe is safe?. *American Journal of Ophthalmology*.

[21] Khanduja S., Kakkar A., Majumdar S., Vohra R., Garg S. (2013). Small gauge vitrectomy: recent update. *Oman Journal of Ophthalmology*.

[22] Delaney Y. M., Oyinloye A., Benjamin L. (2002). Nd:YAG vitreolysis and pars plana vitrectomy: surgical treatment for vitreous floaters. *Eye*.

[23] Schulz-Key S., Carlsson J.-O., Crafoord S. (2011). Longterm follow-up of pars plana vitrectomy for vitreous floaters: complications, outcomes and patient satisfaction. *Acta Ophthalmologica*.

[24] Sebag J. (2011). Floaters and the quality of life. *American Journal of Ophthalmology*.

[25] de Nie K. F., Crama N., Crama M. A. D., Klevering B. J., Boon C. J. F. (2013). Pars plana vitrectomy for disturbing primary vitreous floaters: clinical outcome and patient satisfaction. *Graefe's Archive for Clinical and Experimental Ophthalmology*.

[26] Tan H. S., Mura M., Oberstein S. Y. L., Bijl H. M. (2011). Safety of vitrectomy for floaters. *American Journal of Ophthalmology*.

[27] Shah V. C., Russo C., Cannon R., Davidson R., Taravella M. J. (2010). Incidence of Nd:YAG capsulotomy after implantation of AcrySof multifocal and monofocal intraocular lenses: a case controlled study. *Journal of Refractive Surgery*.

